# Narrowing the gap between analytically convenient and naturalistic stimuli

**DOI:** 10.1167/jov.26.9.4

**Published:** 2026-09-02

**Authors:** Jonathan D. Victor

**Affiliations:** 1 Feil Family Brain and Mind Research Institute, Weill Cornell Medicine, New York, NY, USA

**Keywords:** naturalistic stimuli, systems analysis, efficient coding, visual textures, generative AI

## Abstract

The gulf between mathematically motivated stimuli—such as sine waves and noise ensembles—and images drawn from natural scenes may not be as wide as it appears. For the former, the mathematical properties that support their utility as analytic probes are explicit. For naturalistic stimuli, this is not the case, but they are created by physical processes whose outcomes—at least in principle—heavily constrain their statistics. Images generated computationally occupy a middle ground: Their mathematical properties remain implicit in the rules that generate them, but these rules are simpler than the processes that generate natural images. It is suggested that a greater understanding of the statistics of natural images, while challenging, will enhance their utility as experimental tools and thereby lead to new insights into the design principles of sensory systems.

## Introduction

A main theme of this Special Issue is the choice between naturalistic and artificial stimuli, and the relative merits of these stimulus classes are perhaps the most persistently and passionately debated aspects of experimental design (Felsen & Dan, 2005; Rust & Movshon, 2005; Sharpee, 2013). Discussions on this point often conclude with an agreement to disagree, citing the purpose of the experiment and perhaps the experimenter's philosophy. Here, I suggest greater common ground: that there is fundamental agreement about a core scientific goal, and the naturalistic versus artificial debate reflects an incomplete state of both conceptual and biological understanding, leading to uncertainty as to how the goal is best achieved and a gap that appears wider than it really is. The common goal is a simple one: the need to characterize the behavior of a system across a substantial range of its inputs and to do so using stimuli that drive the normal physiological mechanisms. In attempting to support this claim, I hope that this perspective will provide a common framework for thinking about many stimulus classes, including those that are naturally parameterized in the spatial domain and those that are naturally parameterized in the Fourier domain, as well as noise stimuli, artificial and naturalistic textures, and natural scenes. I also hope that it will suggest directions for new theoretical investigations.

Under some circumstances, stimulus choice is indeed driven by the purpose of the experiment. If the immediate goal is to measure the response to a particular set of naturalistic stimuli, then that stimulus set is the obvious and logical choice. But, if the goal is to understand the neural computations underlying how a system responds in general, the decision is less clear. There the default choice shifts to artificial stimuli, which support stringent tests of a model, including insights into its limitations. Fundamentally, this is often because artificial stimuli are “analytically convenient,” as they constitute a library of simple stimuli that, because of their mathematical properties, can be used to build up more complex ones and to test models.

As it is nearly tautologous that the first goal is well served by selecting natural stimuli, I will attempt to support the claim that the rationales for stimulus choice are convergent by taking the viewpoint of a computational modeler. Here, experimental design requires selection of stimuli from a space with an enormous number of degrees of freedom: in the case of vision, how light intensity varies as a function of two (or three) spatial dimensions, one temporal dimension, and perhaps wavelength. The first step in making this problem tractable is to assume that many of these degrees of freedom are irrelevant—for example, that only a restricted region of space (the receptive field!) and only a particular amount of its immediate past history are important. With such assumptions, along with choosing a spatial resolution and a temporal resolution, the number of degrees of freedom is large but manageable.

However, this restricted space of possible stimuli is far too large to explore by brute force. The standard next step is to make an assumption about the stimulus–response relationship. For neurons in early visual processing, one way to proceed is to assume that linearity is a good approximation: that addition (superposition) of stimuli results in addition of the responses. A related but weaker assumption is that the output results from an additive interaction followed by a pointwise nonlinearity (i.e., one that applies the same transformation at every point in time). Given an assumption of this kind (and we will consider alternatives below), it suffices to probe the input space one dimension at a time, and only a single stimulus on each dimension is necessary (or a few stimuli, if one allows for a pointwise nonlinearity). That is, determining the response of the system to each of the basis vectors in the stimulus space suffices to fully characterize the system. The problem of characterizing responses to the entire stimulus space reduces to the problem of characterizing responses along a set of axes, where the number of axes is equal to the number of dimensions in the space. In principle, the number of such dimensions is infinite, but, with assumptions about temporal and spatial resolution limits, the number is merely very large.

If the system is linear for one basis set, it is linear for all other basis sets. So, an important choice remains: which axes? It makes intuitive sense that an efficient approach is to choose the axes (i.e., the spatiotemporal patterns used to probe the system) so that they are as different as possible from each other, to minimize the redundancy between measurements. Geometrically, this corresponds to axes that are orthogonal, as a correlation between spatiotemporal patterns corresponds to a dot-product between their representations as vectors. But, even within the realm of orthogonal stimulus sets, there are many possibilities: Orthogonality only determines the axes up to rotation. One choice is a stimulus set consisting of spots of light at a chosen spatial resolution, each presented for a duration corresponding to the chosen temporal resolution (the “spacetime” basis set). A second choice is the “Fourier basis,” a stimulus set consisting of sinusoids in space, modulated sinusoidally in time. There are also myriad intermediate choices, including gratings presented as flashes, Gabor functions (Daugman, 1985; Gabor, 1946), Hermite functions, and their generalizations (Victor & Knight, 2003). Each of these choices amounts to choosing a different set of orthogonal (or, in the case of Gabor functions, approximately orthogonal) coordinate axes.

Two general considerations that impact on this choice are illustrated by comparing the spacetime basis with the Fourier basis. One consideration flows from the approximate nature of linearity. Linearity is guaranteed to be violated for stimuli whose magnitude is sufficiently large; conversely, the assumption of linearity is more likely to be useful if stimulus magnitude is limited. On the other hand, the realities of biological and measurement noise mean that one wants stimuli that produce a response that is not close to noise levels. In characterizing a system with a spacetime basis, the spot size and duration must be small, to match the temporal and spatial resolution of the system. But, to achieve a non-negligible response with such a stimulus, the pulse amplitude may have to be large. An abrupt transition from background to a small, bright flash may be the worst-case scenario in terms of what is needed to give the assumption of linearity a chance to be effective.

In contrast, the Fourier basis calls for stimuli that are smoothly modulated in space and time. There is, of course, a tradeoff, as one now has to decide how to sample in the Fourier domain. For many physical systems, characteristics depend smoothly on frequency, so a relatively sparse sampling will suffice. Note also that the strategy of attempting to avoid saturations by seeking a set of stimuli that produce large responses for a given input size amounts to seeking to match the stimuli to the receptive field profile. Often, this corresponds to choices that are intermediate between the spatiotemporal domain and the Fourier domain (Pollen, Nagler, Daugman, Kronauer, & Cavanagh, 1984).

A second, and very different, consideration is that it is often helpful and reassuring to have a direct path from the experimental data to some understanding of the system under study. Both the spacetime strategy and the Fourier strategy have advantages here. The average response to impulses at each spatial location is a direct characterization of the system, and, under the hypothesis of linearity, the superposition of impulse responses suffices to predict the response to any stimulus. Its Fourier transform (i.e., the transfer function), which is directly measured by the responses to sinusoidal stimuli, has convenient analytic properties: the transfer function of subsystems in parallel, in series, or in feedback loops combine by addition, multiplication, and division. Another way that responses to sinusoidal stimuli can provide insight is that the need for nonlinear components is immediately evident in a frequency-domain approach: A strictly linear system will produce outputs only at the stimulation frequency and not at its harmonics.

These considerations may be useful conceptually, but their practical utility fades when one is more than one synapse away from the sensory periphery. Even if approximate linearity holds, neurons may be narrowly tuned; geometrically, this means that there are few directions in stimulus space that lead to responses at all. Nonlinearity only compounds the problem, as a nonlinear neuron may respond very differently to a combination of stimulus components than is predicted by summing the response to each of the components presented in isolation, so characterization of the response along several axes in stimulus space does not characterize the behavior for off-axis points.

Approaches based on noise stimuli—spatiotemporal Gaussian noise, spatiotemporal binary noise, and related strategies—may be viewed as attempts to address this problem, but they typically fall short. Each of these stimulus sets samples a region around the origin, without restriction to a preferred set of axes. Although these approaches have proven effective for characterizing neurons in retina and V1 that have relatively simple nonlinearities and are broadly tuned (Meister & Berry, 1999; Reid, Victor, & Shapley, 1997), they miss critical aspects of neural selectivity in later visual areas, such as V4 (Gallant, Braun, & Van Essen, 1993; Pasupathy & Connor, 2001) and beyond. The reason is straightforward: A noise ensemble broadly samples the space of possible images, but the sampling of any particular image is so infrequent so as to be nonexistent in practical terms. For such neurons and, more generally, for neurons that are highly nonlinear or narrowly tuned, naturalistic stimuli can thus be viewed as providing a shortcut to finding the regions of stimulus space in which a visual neuron will respond and avoiding the much larger volume of stimulus space that is irrelevant.

These considerations concerning choice of a stimulus set apply equally well—if not more so—when the experimenter is recording from large populations of neurons, or describing the response in more abstract terms, such as a response manifold (Langdon, Genkin, & Engel, 2023). The output variable is now more complex or more abstract than a single time series of spikes, but the central issue is the same: How should the set of stimuli be chosen so that the full range of neural behavior can be observed?

I expect that the comments up to this point are non-controversial, perhaps even obvious. But now I would like to take a more speculative viewpoint: that naturalistic stimuli will ultimately turn out to have much more in common with “constructed” stimulus sets than we currently imagine. It's just that we do not yet have the insights to recognize this.

The first claim is that we may be far from what is achievable in terms of a mathematical characterization of naturalistic stimuli. We know that naturalistic stimuli are a miniscule subset of all possible stimuli, and we have some insights as to what this subset is, but it appears quite incomplete. For example, naturalistic stimuli have distinctive statistics (e.g., the 1/*f*^2^ power spectrum) (Field, 1987)—but images created with this power spectrum and no other constraints do not in any way look natural. At least part of the reason is that naturalistic stimuli have other statistical properties not captured by the power spectrum for example, local image statistics (Hermundstad et al., 2014; Tkacik, Prentice, Victor, & Balasubramanian, 2010) and correlations across frequency bands (Schwartz & Simoncelli, 2001). It is a difficult mathematical problem to sample randomly from images with statistical constraints on the spectra and higher-order quantities, so it is unclear whether these relatively simple statistical constraints suffice to account for the “natural” subset of all possible stimuli. Intuitively, a large number of degrees of freedom remain, but, surprisingly, work by Wakita and Motoyoshi (2026) has shown that a wide range of naturalistic textures can be captured by as few as 16 latent variables. This emphasizes the remaining gap between current mathematical characterizations of natural images, and empirical evidence.

It is, of course, risky to claim that an insight is achievable, without providing a path to achieve it, so here is a thought experiment to explain my optimism. Imagine being a vision scientist at a time in which the standard mathematical functions were just the polynomials, and one was tasked with describing a movie of an object on a spring. Basic physics yields the differential equation that governs its motion: Acceleration, which is proportional to force, is a linear function of position. Because acceleration is the second derivative of position, one could then calculate the motion path numerically. Now, it is also well known that the solutions to this differential equation are simply the sines and cosines of an appropriate frequency. In the absence of a compact notation for trigonometric functions, one could be forgiven for considering these solutions to be awkward objects, as they are not readily expressed in terms of the familiar elements (i.e., the polynomials) and hence might be difficult to work with. But, importantly, the solutions to the equations of motion have some really nice properties—periodicity and orthogonality—and these properties can be demonstrated directly from the motion equation, without ever producing a simple formula for its solutions. This changes how the solutions are viewed: from awkward objects known only numerically to indispensable elements of mathematical analysis.

Two approaches to the study of visual texture provide for a transition from a thought experiment to real experiments. The well-known texture-synthesis procedure of Portilla and Simoncelli (2000) generate naturalistic images whose statistical properties are implicit in the iterative algorithm that generates them. But, even without an explicit characterization of these statistics, they are useful tools in characterizing the computations carried out in early visual areas (Freeman, Ziemba, Heeger, Simoncelli, & Movshon, 2013).

In our laboratory, a set of texture-synthesis algorithms (Victor & Conte, 2012), loosely inspired by the dynamics of a physical system of interacting spins (Pickard, 1980), generates a high-dimensional space of visual textures. For textures in which the interactions have simple forms (pairwise and some higher-order cases), their correlation structure can be described with standard exponentials (Victor & Conte, 2022). But, for more complex interactions, no such simple representation appears possible. Nevertheless (Victor & Conte, 2012), these textures have a “maximum entropy” property that follows directly from the texture generation procedure; that is, they are as random as possible given the local rules that generate them. The maximum-entropy property allows a normative model to connect perceptual thresholds measured with these textures to the statistics of natural images (Hermundstad et al., 2014; Tesileanu et al., 2020; Tkačik, Prentice, Victor, & Balasubramanian, 2010).

Natural scenes and the images they form are, of course, quite complex, as they are driven by multiple kinds of processes that act at multiple scales, and we do not fully understand them. Nevertheless, even in this scenario, insights are possible. As an example, the characteristic 1/*f*^2^ spectrum of natural scenes can be derived from a simple symmetry argument, along with the heuristic that a natural scene consists of occlusive, discrete objects. When one “steps into” a natural scene, another natural scene results; some objects are now behind the viewer, and distant objects become closer, subtending a larger angle. The 1/*f*^2^ spectrum follows from the observation that the resulting *statistics* cannot change; it is the only spectrum that has this property. Interestingly, a variation of this argument applies to scenes composed of partially transparent objects that fill space and are viewed at a fixed distance (as in some medical radiographs, such as mammograms); here, the spectrum changes to 1/*f*^3^ (Burgess, Jacobson, & Judy, 2001).

In the thought experiment of a movie of an oscillating weight, the key mathematical properties of the motion (e.g., the periodicity and orthogonality of sines and cosines) can be derived from the generative process, without producing explicit solutions. In the texture-synthesis algorithms of Victor and Conte (2012), this holds for the maximum-entropy property, and, in the natural-scenes example, it holds for the power spectrum. In the former case, these mathematical properties underlie the utility of these stimulus sets as analytic tools; in the latter, it enables fundamental insights about the design principles of early vision (Field, 1987; Kuang, Poletti, Victor, & Rucci, 2012). The hope is that these examples represent starting points for further progress, rather than bounds. If so, then strategies that focus on the physical processes that generate natural images will prove similarly fruitful, leading directly to identification of their distinctive mathematical properties without a requirement for explicit synthesis.

Interestingly, we are already comfortable with the use of stimulus sets defined by computational generative processes. Textures typically fall into this category. A set of moments or distributional properties of an image ensemble are specified, along with a procedure for creating exemplars. The creation procedure is based on the heuristic of choosing a “random” example of an image drawn from the ensemble. In some cases, the procedure is an explicit sequence of steps: phase randomization (Freeman et al., 2013) or a Markov process (Julesz, 1962; Victor & Conte, 2012) and indeed leads to stimuli that have convenient mathematical properties. In others, the mathematical properties of the outcome are less clear. This includes generative processes that are a one-pass sequence of steps (Skriabine, Shinn, Picard, Harris, & Carandini, 2026), as well as those that are iterative (e.g., the procedure of Portilla and Simoncelli (2000), and later variants) (Freeman & Simoncelli, 2011; Lieber, Oleskiw, Palmieri, Simoncelli, & Movshon, 2026).

More elaborate generative methods taking their algorithms from machine vision have also proven their worth in revealing general features of tuning properties in V1 and V4 (Franke et al., 2026), in defining the selectivity of inferotemporal neurons (Ponce et al., 2019) and in identifying causal relationships between activity in inferotemporal cortex and perception (Shahbazi, Ma, Pernus, Scheirer, & Afraz, 2024). These generative processes are distinct from the generative processes that underlie real-world images, and the images that they generate are not natural but (to human observers) they have recognizable, naturalistic features, although they are not nameable. The images activate visual neurons deep within the visual system quite well—indeed, demonstrably better than real-world natural stimuli (Bashivan, Kar, & DiCarlo, 2019; Ponce et al., 2019). Moreover, these stimuli are particularly incisive stress tests of network models of V4 neurons, as they reveal the ability of models to predict how such stimuli can drive neural activity outside the range that is observed in response to natural images (Bashivan et al., 2019), as well as substantial discrepancies between responses of real V4 neurons and the models (Ren & Bashivan, 2024). One may even speculate that the quantitative differences revealed by such stimuli are reflections of more fundamental qualitative differences (e.g., “off-manifold” versus “on-manifold” activity patterns) (Oby et al., 2025), when ensemble responses are analyzed by geometric means (Langdon et al., 2023).

Physical processes are not the same as the generative processes used in machine vision, but the visual system does not require that stimuli are strictly natural. Visual systems are well adapted to our visual world, but they are not so narrowly tuned to “natural” images that they cannot make sense of things that are novel (Schmittwilken, Haverkamp, & Maertens, 2025). The visual system—like many other sensory systems—is shaped by evolution and development to work efficiently for inputs that are natural (Barlow, 1961), but it is enough of a generalist to behave usefully for inputs unlike those that have been previously encountered. The strategies that it uses to achieve this performance are as yet unclear, and uncovering them may eliminate the apparent gap between natural and computationally convenient stimuli.

In sum, I am suggesting that the gap between analytically convenient stimuli and naturalistic ones may not be as wide as it appears. Naturalistic stimuli are created by physical processes whose outcomes, at least in principle, are fully susceptible to a mathematical description. At present, our characterizations of natural stimuli and their statistics—although clearly important for understanding visual processing—are far from complete. Bridging this gap is a challenge for future work, and even narrowing it is likely to lead to new insights into the design principles of sensory systems.
